# Quantum cascade lasers grown on silicon

**DOI:** 10.1038/s41598-018-24723-2

**Published:** 2018-05-08

**Authors:** Hoang Nguyen-Van, Alexei N. Baranov, Zeineb Loghmari, Laurent Cerutti, Jean-Baptiste Rodriguez, Julie Tournet, Gregoire Narcy, Guilhem Boissier, Gilles Patriarche, Michael Bahriz, Eric Tournié, Roland Teissier

**Affiliations:** 1 IES, University of Montpellier, CNRS, Montpellier, France; 2 Centre for Nanosciences and Nanotechnology, CNRS, University Paris-Sud, Marcoussis, France

## Abstract

Technological platforms offering efficient integration of III-V semiconductor lasers with silicon electronics are eagerly awaited by industry. The availability of optoelectronic circuits combining III-V light sources with Si-based photonic and electronic components in a single chip will enable, in particular, the development of ultra-compact spectroscopic systems for mass scale applications. The first circuits of such type were fabricated using heterogeneous integration of semiconductor lasers by bonding the III-V chips onto silicon substrates. Direct epitaxial growth of interband III-V laser diodes on silicon substrates has also been reported, whereas intersubband emitters grown on Si have not yet been demonstrated. We report the first quantum cascade lasers (QCLs) directly grown on a silicon substrate. These InAs/AlSb QCLs grown on Si exhibit high performances, comparable with those of the devices fabricated on their native InAs substrate. The lasers emit near 11 µm, the longest emission wavelength of any laser integrated on Si. Given the wavelength range reachable with InAs/AlSb QCLs, these results open the way to the development of a wide variety of integrated sensors.

## Introduction

The 20th Century has seen unparalleled success of the silicon-based microelectronics industry, whereas the 21st Century is witnessing the explosion of photonics. Silicon photonics lies at the convergence of both fields and promises to be the next disruptive technology for integrated circuits^1^. This however requires that the whole set of optoelectronics functions be integrated onto a Si platform. While various Si-based modulators and photodetectors have already been demonstrated^2–5^, integrated light sources have for long remained a challenge^6^. Silicon-based sources would straight away bridge the gap but the indirect bandgap of Si or Ge is a severe limitation and, in spite of unquestionable advances^7–10^, such devices will not outperform in the foreseeable future their III-V semiconductor counterparts which remain the most efficient semiconductor laser technology. Much work has thus been devoted in the last decade to integrating III-V laser diodes on Si platforms for telecom applications. Impressive results have been achieved in the visible to near infrared wavelength range by both heterogeneous integration, where III-V materials are bonded to silicon^11–14^, and direct epitaxial growth of III-V laser diodes on Si substrates^15–20^. In parallel, extending silicon photonics toward the mid-infrared (MIR) wavelength spectral region (2–20 µm) has emerged as a new frontier^21^. Indeed, most molecules exhibit absorption fingerprints in the MIR range^22^, which is of crucial interest for societal applications such as health diagnostics, detection of biological and organic compounds, monitoring of toxic gases or of greenhouse gas emission, to name but a few. MIR Si photonics could thus lead to integrated, compact, cost-effective, smart spectroscopy instruments^21,23^. Several groups have already demonstrated MIR platforms at different wavelengths using various Si-based technologies^24–29^. Frequency combs have been shown spanning near- to mid-infrared wavelengths on silicon^30–32^. The availability of integrated MIR light sources is now the remaining key element to be unlocked for developing a large portfolio of sensing systems.

We have described the epitaxial integration of GaSb-based laser diodes emitting in the continuous wave (cw) regime near room temperature (RT) at 2 µm^33^. Lasing near 2.2 µm at cryogenic temperatures has been obtained under optical pumping in direct bandgap GeSn layers epitaxially grown on thick virtual Ge/Si substrates^34^. These two publications are the only reports of the epitaxial integration of a MIR semiconductor laser on Si. More recently, heterogeneously integrated InP-based type II laser diodes have been demonstrated to operate in pulsed mode at RT near 2.4 µm^35^. Finally, InP-based quantum cascade lasers (QCLs) have been heterogeneously integrated on Si and laser emission in the pulsed regime has been achieved at room temperature near 4.8 µm^36^. This remains the only report of the integration of a QCL on Si and no QCLs directly grown on Si have been reported to date. The QCL technology however exhibits a number of advantages making it extremely attractive for developing integrated MIR sensing systems: it allows emission from ∼3 µm up to the THz wavelength range by a proper design of the laser structure^37,38^, this is the most energetically efficient laser technology^39,40^, it supports frequency combs^41^. Integrating QCLs on Si is thus a crucial challenge on the way to smart sensing systems.

Due to the high conduction band offset and the small electron effective mass the InAs/AlSb material family is very attractive for use in QCLs. With these materials we have demonstrated lasers with record performances, such as QCLs operating in the cw regime at RT above 15 µm – the longest RT cw emission wavelength of semiconductor lasers^42^, and pulsed QCLs operating above RT at 20 µm – the longest emission wavelength of semiconductor lasers at RT^43^. In this work, we present InAs/AlSb QCLs grown by molecular beam epitaxy (MBE) on a Si substrate and we compare them with similar devices grown side-by-side on their native InAs substrate. We suppose that modifications of the III-V QCL performances due to the growth on a nonpolar group-IV element substrate with a large lattice mismatch do not depend much on the emission wavelength of the device. For this demonstration we have chosen to fabricate a laser emitting at 11 µm, a central wavelength in the MIR.

## QCL design and epitaxial growth

The active zone of the QCL designed to emit at 11 µm was based on a design with vertical transitions in four coupled quantum wells. It consisted of 40 repetitions of the following layer sequence: **21**/96/**2.8**/76/**2.9**/73/**3**/70/**6**/64/**7**/62/**7**/58/**9**/57/**14**/56/**17**/55, in Å and starting from the injection barrier, where AlSb layers are in bold and the Si-doped layers (n = 4 × 10^16^ cm^−3^) are underlined. The total electron sheet density in the structure, taking into account a typical level of residual doping of n = 5 × 10^15^ cm^−3^ in our materials, is considered to be 8 × 10^10^ cm^−2^ per period. A plasmon enhanced dielectric waveguide of the laser was formed by 2-µm-thick cladding layers made of n  -InAs doped with Si to 2 × 10^18^ cm^−3^. In order to reduce the overlap of the guided mode with the absorbing doped material and to minimize the propagation losses the active zone with a total thickness of 3 µm was separated from the cladding layers by 2.5-µm-thick undoped InAs spacers. The electromagnetic modelling of the guided modes, using a finite element solver, gives an overlap of the fundamental mode with the active region Γ = 56% and the waveguide loss ${\alpha }_{w}$ = 3 cm^−1^ (not including losses in the active region).

The structure was grown simultaneously on two 2″ substrates. The first one was a n-InAs (100) substrate usually employed to grow InAs/AlSb QCLs. Devices fabricated from this wafer were used as a reference. The second substrate was a (100) silicon substrate with a 6° miscut towards the [110] direction. The 6° misfit was chosen to limit the formation of anti-phase domains appearing during the growth of III-V materials on non-polar group IV substrates^44^.

**Figure 1:**
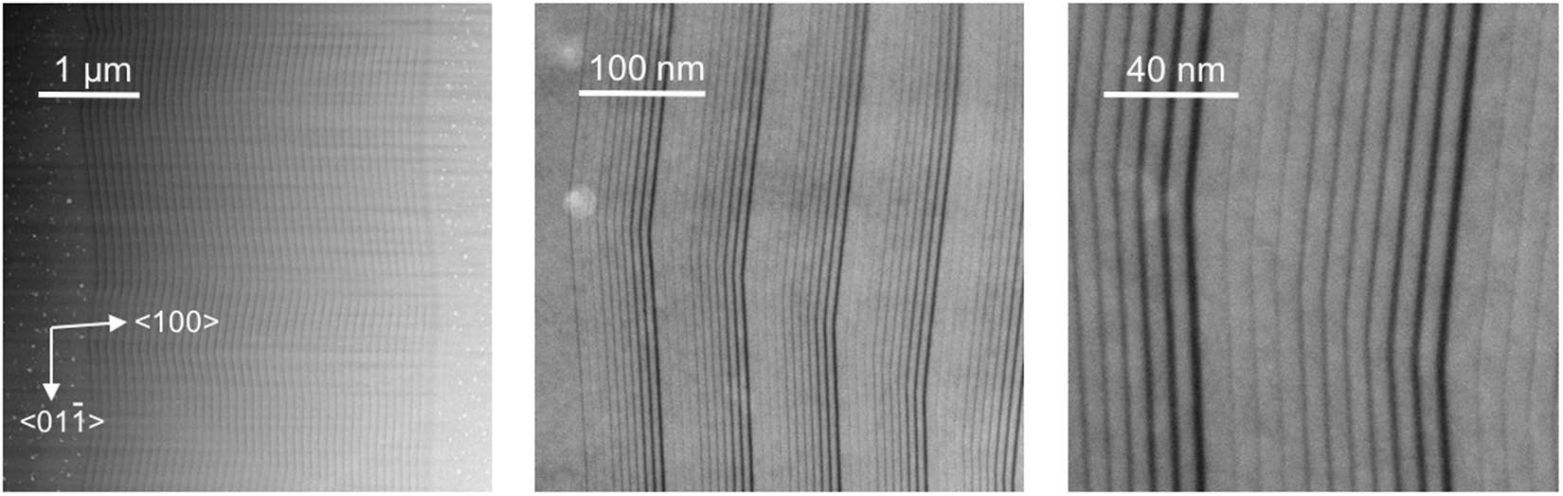
High angle annular dark field STEM images of the QCL active zone grown on Si at different magnifications. Dark regions correspond to AlSb layers. The substrate is on the left.

Figure 1 presents scanning transmission electron microscopy (STEM) images of the QCL active zone grown on Si taken in the high angle annular dark field regime (HAADF) at different magnifications. These pictures show that the growth was not planar, the epitaxial layers exhibit a wavy behavior. A characteristic lateral size of these undulations is about 2–3 µm. Such “long” period cannot be explained by the steps formed by deviation of the growth plane from the exact crystallographic direction. The 6° miscut of the used Si substrate corresponds to 3-nm-long atomic steps. Step bunching aggregating many individual steps could in principle result in such spatial modulation but it is generally accompanied by abrupt periodic shifts of the position of epitaxial layers, which is not observed here. All layers are perfectly continuous along the active zone and even the thinnest AlSb barriers are clearly visible at high magnification. We explain the observed non-planarity of the growth by a modulation of the growth rate by the strain field around the dislocations arising from relaxation of the high lattice mismatch (∼11.5%) between the Si substrate and the InAs-based structure. The dislocation density corresponding to the characteristic size of the spatial undulations can be estimated to be in the range (1–3) × 10^7^ cm^−2^. The main part of the thickness modulation was accumulated during the growth of the thick InAs layers beneath the active zone. As a result, the thickness of the epitaxial layers is not constant along the growth plane because of these spatial fluctuations of the growth rate. The variation of the layer thickness estimated from the STEM images in the active zone reached maximum values of 9% for individual QWs and 7% for the total active zone thickness (*h*_*AZ*_). The standard deviation of *h*_*AZ*_, extracted from a 10 µm long lateral section, is 1.8%.

## Device fabrication

**Figure 2:**
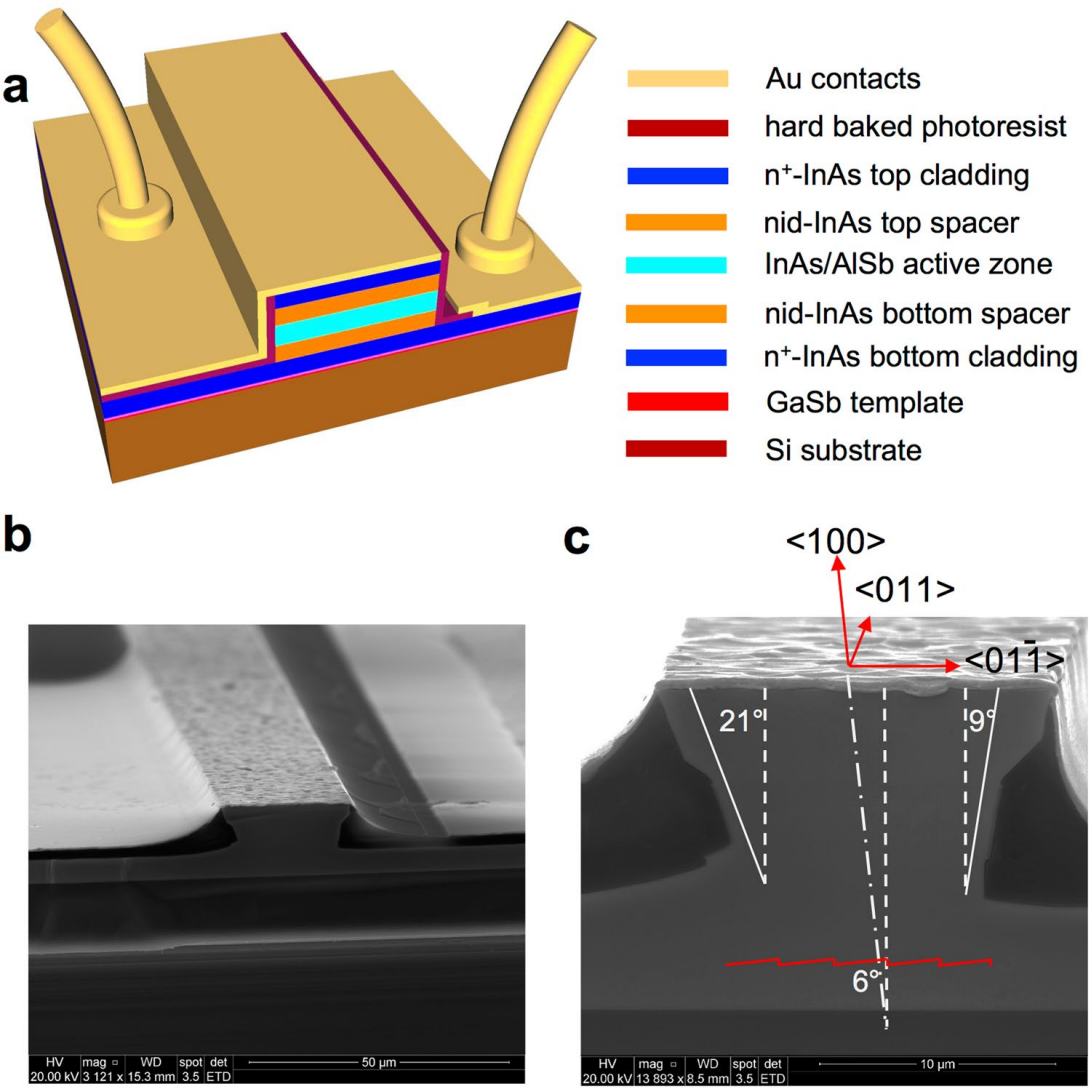
Schematic and SEM photographs of the fabricated lasers. (**a**) Schematic of ridge lasers fabricated on the Si substrate. Different materials and layers are denoted in the order of their appearance in the structure by the color code shown in the right. (**b**) SEM image of the fully processed wafer. Contact pads correspond to the scheme presented in Fig. 1a. (**c**) SEM image of the cleaved facet of the laser demonstrating an asymmetric shape of the ridge formed by anisotropic wet etching of the misoriented structure. Crystallographic directions and a schematic of atomic plane steps induced by the miscut of the substrate are also shown.

The as-grown wafers were processed into ridge lasers using wet etching and conventional UV photolithography. The ridge width *w* varied between 10 and 22 µm. Electrical insulation was provided by hard baked photoresist. After processing, the substrates were thinned down to 100–150 µm (InAs substrate) or 50 µm (Si substrate) by mechanical polishing. On both wafers the laser ridges were etched down to the bottom n^+^-InAs cladding layer. In the sample grown on InAs, electrical contacts were fabricated on the top of the ridges and on the back side of the substrate. In the structure grown on Si, both contacts were formed on the top of the wafer. The contacts to the devices were made using non-alloyed Ti/Au metallization. The schematic of ridge lasers fabricated on the Si substrate is shown in Fig. 2a and a scanning electron microscope (SEM) image of the processed wafer is presented in Fig. 2b. The first cladding layer served as a bottom electrical contact (right part of the ridges shown in Fig. 2).

The top contact pad was placed on the opposite side of the ridges. The ridges were oriented along the <011> direction and the atomic plane steps of the epitaxial layers produced by the misorientation of the substrate formed a staircase running perpendicularly to the ridge axis, which is shown schematically in Fig. 2c. The ridge profile was asymmetric due to the anisotropic wet chemical etching of the misoriented structure. The asymmetry of the ridge shape corresponds to the 6° miscut of the Si substrate. For both types of lasers, the ridge width was measured on the cleaved facet in the middle of the active zone clearly visible due to the etching selectivity. Laser chips with cleaved Fabry-Perot resonators and uncoated facets were soldered epi-side up onto copper heatsinks using indium. No special selection was made to choose the devices for this study.

## Results

**Figure 3:**
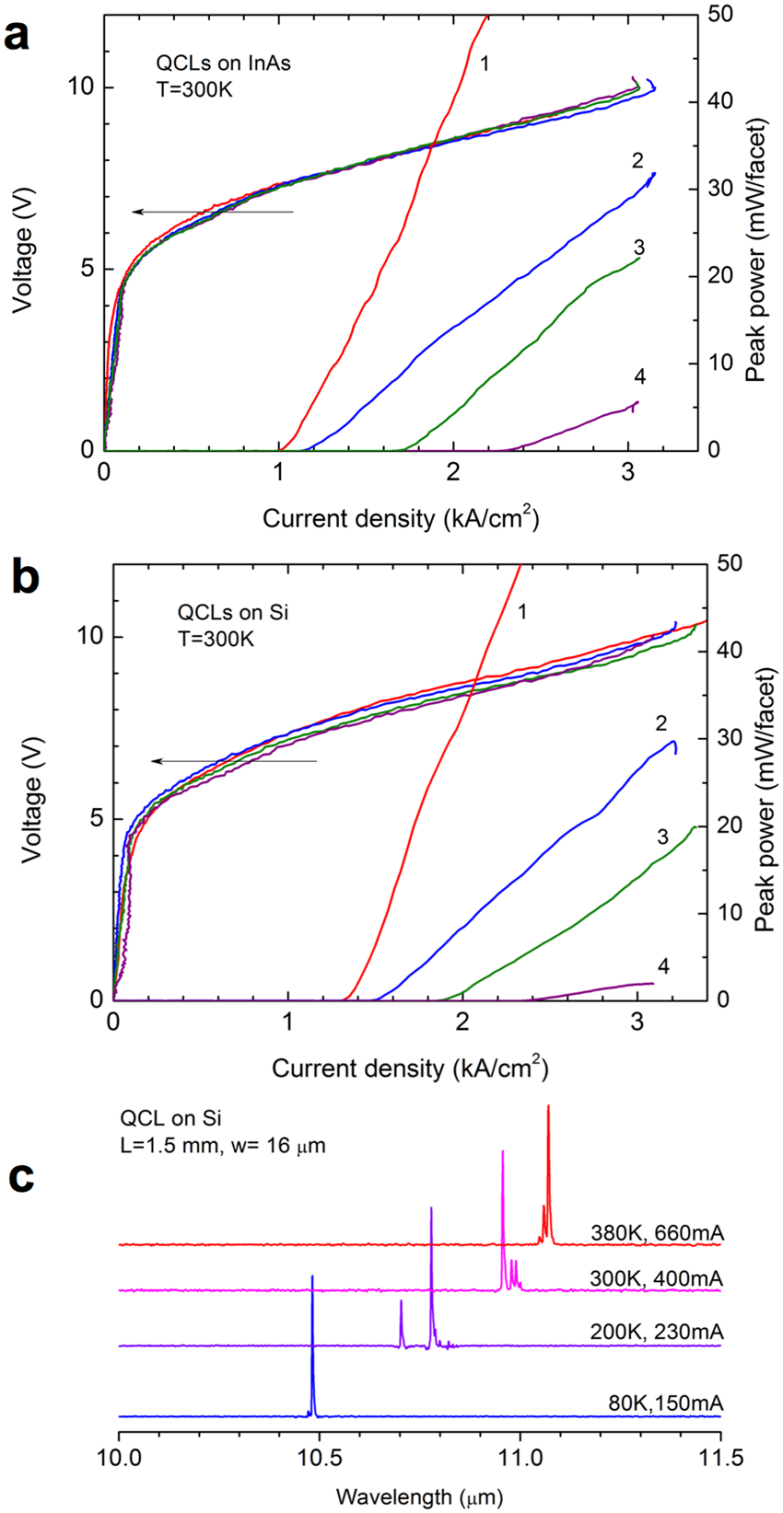
Characteristics of the studied QCLs. L – resonator length, w – ridge width. (**a**) Voltage-current and light-current characteristics of QCLs grown on InAs. 1 – L = 3.5 mm, w = 21 µm; 2 – L = 2.3 mm, w = 14 µm; 3 – L = 1.2 mm, w = 17 µm; 4 – L = 0.7 mm, w = 17 µm. (**b**) Voltage-current and light-current characteristics of QCLs grown on Si. 1 – L = 3.0 mm, w = 20 µm; 2 – L = 1.5 mm, w = 16 µm; 3 – L = 1.15 mm, w = 15 µm; 4 – L = 0.6 mm, w = 14 µm. (**c**) Emission spectra of a QCL grown on Si measured at different temperatures.

QCLs grown on InAs exhibited RT pulsed threshold current densities *J*_*th*_ as low as 1.03 kA/cm^2^ and peak optical powers exceeding 100 mW/facet for the longest, 3.5-mm-long, devices. The threshold current density increased up to 2.4 kA/cm^2^ in the shortest, 0.6-mm-long lasers. Voltage-current and light-current characteristics of the lasers grown on InAs are shown in Fig. 3a. QCLs grown on Si also showed similar high performances. The 3-mm-long devices demonstrated *J*_*th*_ only 30% higher than that of the lasers grown on InAs (Fig. 3b). Moreover, in the short devices *J*_*th*_ was the same or even lower than in the reference QCLs. The slope of the light-current curves was also comparable in both types of lasers. The maximum available current density *J*_*max*_, corresponding to the loss of alignment of the injector and the upper level of the lasing transition at high electric fields, was (3.1–3.4) kA/cm^2^ in the QCLs grown on Si and (3.0–3.1) kA/cm^2^ in the reference lasers. The higher values of *J*_*max*_ can be due to the error in measuring the effective width of the asymmetric ridges, which would result in underestimation of the device surface. It should be noted that in this case *J*_*th*_ of the lasers grown on Si would be slightly overvalued. Another reason could be a higher doping level in the structure grown on the misoriented Si substrate. Both types of lasers emitted near 11 µm at room temperature. The evolution of emission spectra with temperature is shown in Fig. 3c for a laser grown on Si. The QCL emission wavelength typically increased from 10.5 µm at 80 K to 11.1 µm at 380 K.

**Figure 4:**
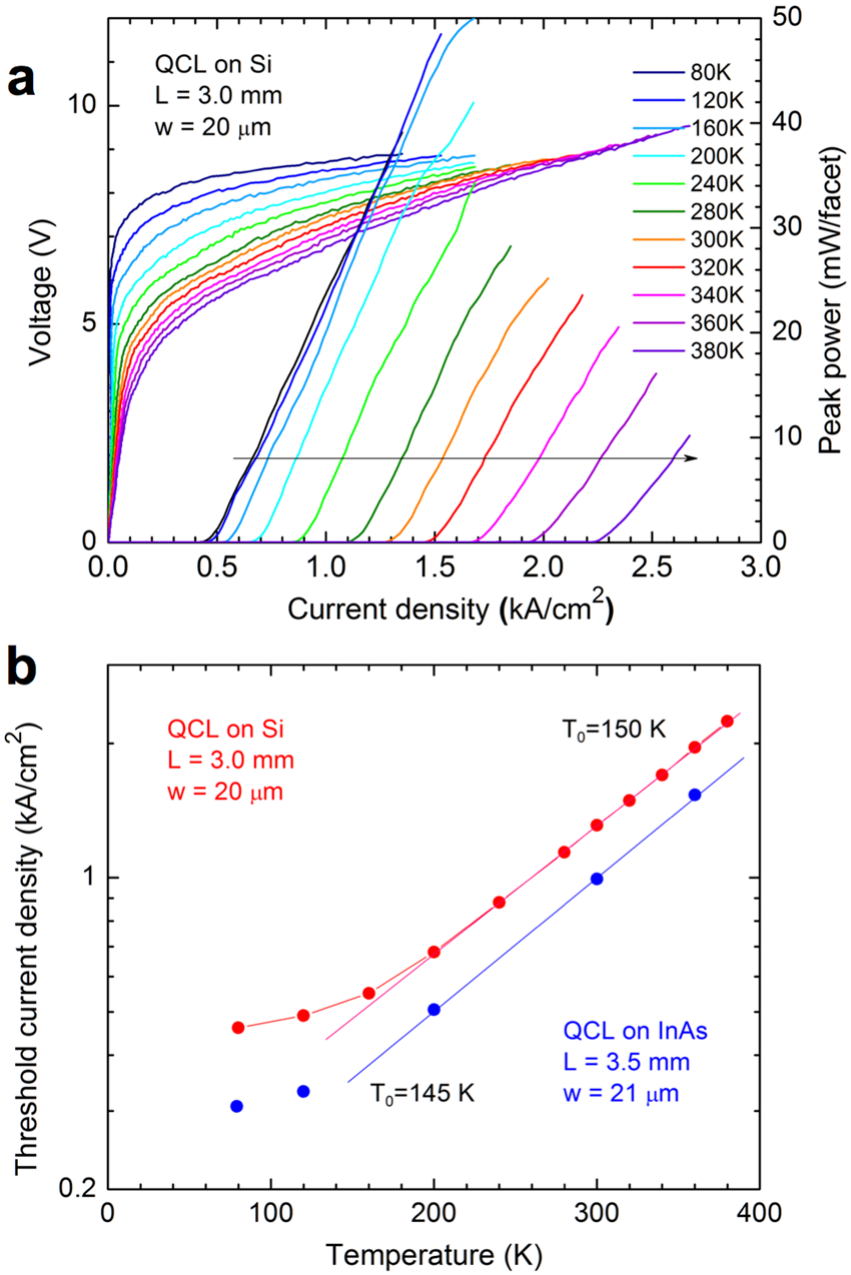
Characteristics of the studied QCLs at different temperatures. (**a**) Voltage-current and light-current characteristics of a QCL grown on Si measured between 80 and 380 K. (**b**) Threshold current density as a function of temperature of QCLs grown on Si and on InAs.

Figure 4a presents voltage-current and light-current characteristics of a 3-mm-long laser grown on Si measured between 80 and 380 K. The temperature dependence of *J*_*th*_ for this laser is shown in red in Fig. 4b. The threshold current density increased slowly with temperature between 80 and 160 K whereas above 180 K its temperature dependence was exponential with a characteristic temperature *T*_0_ = 150 K. Figure 4b shows also the data for a reference laser (in blue). They demonstrated a similar temperature behavior but *J*_*th*_ increased however a little faster in the exponential part of the curve, with a characteristic temperature *T*_0_ = 145 K.

**Figure 5:**
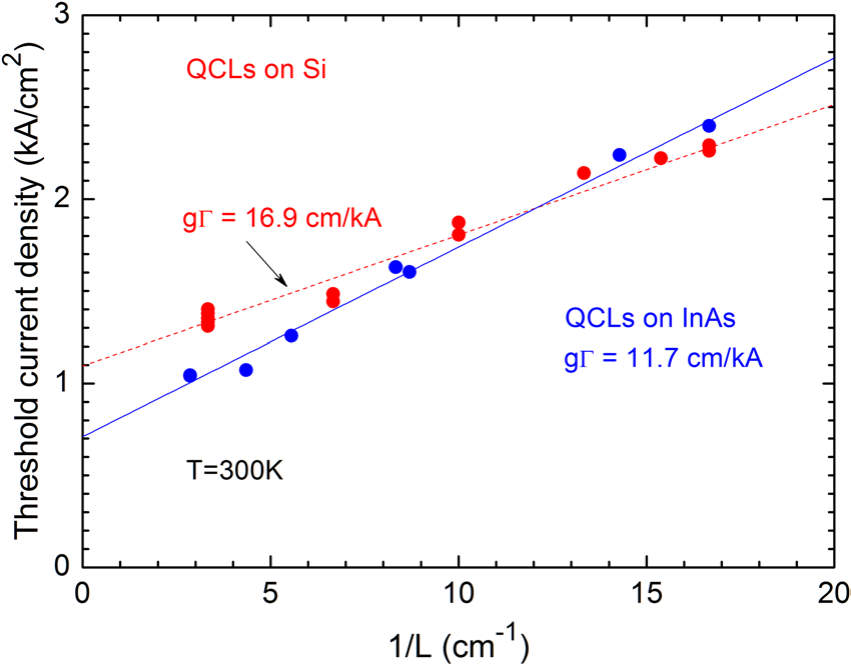
Threshold current density of the studied QCLs as a function of reciprocal resonator length. The plotted values are normalized for the ridge width of 20 µm.

We measured a large number of devices with different dimensions. The threshold current density of the wide lasers decreased by about 20% compared with narrow devices when the ridge width varied in the range of 10–20 µm. The higher *J*_*th*_ in narrow QCLs is due to a larger overlap of the optical mode with absorbing dielectric on the mesa walls^44^. We consider that this, quite weak, effect is not further influenced by the different shape of the ridges in the lasers grown on Si and InAs. Taking into account this width dependence, the room temperature threshold current density of the lasers normalized for *w* = 20 µm is plotted in Fig. 5 as a function of the reciprocal cavity length 1/*L*. This dependence can be analyzed using the following equation^45^:

1 $${J}_{th}={J}_{tr}+\frac{({\alpha }_{w}+{\alpha }_{m})}{{\rm{\Gamma }}g}={J}_{tr}+\frac{{\alpha }_{w}}{{\rm{\Gamma }}g}\,-\,\frac{\mathrm{ln}(R)}{{\rm{\Gamma }}g}\,\frac{1}{L}$$

where *J*_*tr*_ is the transparency current of the active region, ${\alpha }_{w}$ is the optical absorption in the waveguide confining layers, ${\alpha }_{m}$ is the mirror loss depending on the resonator length, *g* is the differential gain and ${\rm{\Gamma }}$ is the fundamental guided mode overlap with the active zone.

The facet reflectivity *R* calculated using the Fresnel formula yields *R* = *0*.2*9* for the given waveguide. The slope of the *J*_*th*_ (1/*L*) dependence gives the differential modal gain, ${\rm{\Gamma }}g$ = 11.7 cm/kA for QCLs grown on InAs (Fig. 5). Taking into account the calculated waveguide loss ${\alpha }_{w}$ = 3 cm^−1^ one can obtain ${J}_{tr}$ = 0.45 kA/cm^2^. This value, about 40% of the threshold current densities, is due to the thermal population of the bottom level of the laser transition and to leakage currents. The relatively weak contribution of ${J}_{tr}$ into the total ${J}_{th}$ evidences that both the QCL design and the device processing have been well implemented in these lasers. The data for lasers grown on Si, also presented in Fig. 5, are more dispersed but it is clearly seen that the threshold current densities are comparable with those of the devices grown on a native InAs substrate. More surprisingly, the differential gain is higher (${\rm{\Gamma }}g$ = 16.9 cm/kA), resulting in lower ${J}_{th}$ for shorter lasers as compared with the reference InAs wafer. In Fig. 5 the larger intercept at 1/L = 0, due to the term ${J}_{tr}+\frac{{{\rm{\alpha }}}_{{\rm{w}}}}{{\rm{\Gamma }}g}$ in Equ. (1), may indicate stronger optical loss in the whole waveguide or more significant current leakage because of the likely higher doping mentioned above, due to the growth on a misoriented substrate, which, in turn, explains the somewhat higher ${J}_{th}$ in long devices grown on Si. The additional optical loss can also be due to the high density of crystalline defects. It should be noted, that the quite small deviation of the experimental points in Fig. 5 from the corresponding linear fits can be considered as an evidence of the consistency of the obtained results.

## Discussion

The high performance of the QCLs grown on Si is quite unexpected, especially taking into account the worse crystalline quality as compared with the wafer grown on InAs. To analyze possible explanations of this finding, let us consider the usual equation for the QCL gain coefficient^46^:

2 $$g={\tau }_{3}(1-\frac{{\tau }_{2}}{{\tau }_{32}})\frac{{e}^{2}\hslash {f}_{32}}{{\varepsilon }_{0}nc2{\gamma }_{32}{L}_{p}{m}_{0}}$$

where ${\tau }_{3}$, ${\tau }_{2}$, ${\tau }_{32}$, ${f}_{32}$ are the lifetimes and the oscillator strength characterizing the 3 to 2 lasing transition, e*, m*_*o*_ – the electron charge and effective mass, respectively, *n* – the effective refractive index of the optical mode, *L*_*p*_ – the length of one period of the QCL active zone, *c* – the speed of light, and *2γ*_32_ – the width of the gain spectrum. In this equation only *γ*_32_ and the lifetimes can be influenced by the crystalline quality of the structure. Considering the observed fluctuations of the layer thickness (Fig. 1) one can expect a broadening of the gain spectrum in the QCLs grown on Si. Typically, 2*γ*_32_ is of the order of 10% of the transition energy. To some extent, the relative wavelength variation can be considered as proportional to the variation of the thickness of quantum wells in the QCL active region. Hence, the observed average inhomogeneity of the layer thickness (Fig. 2), estimated above to be +/−1.8% should result in a 36% broadening of the initial gain curve and a corresponding decrease in the QCL gain. It is however reasonable to suppose that the thickness of AlSb barriers is also modulated, in phase with the InAs fluctuations. The cumulative effect of the thickness variation of wells and barriers results in a much weaker change in the transition energy, because thicker barriers reduce the transition wavelength. We estimated that the 3.6% synchronous change in the thickness of all layers in the structure results in a <1% variation in the emission wavelength. Hence the observed average thickness fluctuations should only result in a small increase of the gain width not exceeding 10%. To summarize, we believe that thickness fluctuations in the active zone certainly decrease the gain of QCLs grown on Si but this influence is weak because of the opposite effect of the well and barrier fluctuations.

The most surprising finding of this work is a larger differential gain in the QCLs grown on Si compared with lasers grown on a natural InAs substrate. Considering again Equ. (2), the higher gain could be explained by changes in the intersubband lifetimes. The contribution to lifetime ${\tau }_{3}$ due to LO-phonon emission is calculated to be 0.42 ps in the investigated structure. In general, the very short lifetimes make QCLs insensitive to many parasitic recombination mechanisms existing in other types of semiconductor lasers. Among the parasitic paths that can affect the QCL operation, the interface roughness scattering is however an important one. The efficiency of this mechanism depends on the effective roughness height *Δ* and on the characteristic lateral size of these fluctuations, the correlation length *Λ[]*^47^. Typical parameters that satisfactory describe the available experimental data on InAs/AlSb QCLs grown on InAs substrates are the following: *Δ* = 0.03 nm, *Λ* = 4.5 nm. These values show that the interface scattering is due to spatial fluctuations at a much smaller scale than the micrometer-scale features related to dislocations discussed above and shown in Fig. 1. The ${\tau }_{3}$ lifetime drops to 0.30 ps if the interface scattering is taken into account using these parameters. It is reasonable to assume that the presence of the short atomic steps, due to the growth on a 6° misoriented Si substrate, can significantly affect the lateral size of the interface roughness. More precisely, it can decrease the effective roughness of the interfaces by favoring a smoother step-flow growth thus reducing the interface scattering rate and, as a result, increasing ${\tau }_{3}$. Such mechanism could be responsible for the larger differential gain in these devices.

## Conclusion

In summary, we demonstrated the first quantum cascade lasers directly grown on a Si substrate. The RT threshold current density of these InAs/AlSb QCLs is as low as 1.3 kA/cm^2^ in 3-mm-long devices with a cleaved Fabry-Perot resonator. The lasers operated near λ = 11 µm in pulsed mode and the maximum operation temperature exceeds 380 K. These QCLs exhibited high performances comparable with those of similar devices grown on their native InAs substrate. The absence of degradation despite the poorer crystalline quality is explained by the natural insensitivity of QCLs to crystalline defects. Quite the opposite, a higher differential gain is observed in these lasers, that could be due to modifications of the interface scattering through the influence of short atomic steps formed because of the 6° miscut of the employed Si substrate. Given the wide wavelength range reachable with InAs/AlSb QCLs, this opens the way to the development of a large portfolio of integrated sensors in the whole MIR.

## Methods

### MBE growth

A RIBER 412 solid-source MBE system equipped with valved cracker cells for both Sb and As was used to grow the QCL material. Growth rates were set to 1 ML/s for InAs and 0.33 ML/s for AlSb. Both materials were grown at a V/III flux ratio of about 2.

### Si substrate preparation

Prior to epitaxy the Si substrate was prepared by applying both *ex-situ* and *in-situ* procedures described earlier in detail^48^. The growth was initiated by depositing 4 monolayers AlSb directly on the Si substrate at 450 °C, followed by the growth of the GaSb buffer while ramping the substrate temperature up to 500 °C^49^. After 1-µm GaSb, the temperature was ramped down to 450 °C in order to grow a 200-nm-thick InAs layer. After the template completion, the sample was taken out of the MBE system to be mounted side-by-side with an InAs substrate on a multiwafer Mo-bloc. The QCL growth was then performed exactly in the same conditions on the InAs substrate and on the III-V-on-Si template.

### STEM measurements

The STEM images were obtained with the FIB preparation in the <110> zone axis parallel to the steps related to the vicinality of the substrate. HAADF-STEM was performed on a spherical aberration corrected Titan Themis microscope operated at 200 kV.

### Device characterization

The fabricated devices were mounted in a LN_2_ flow cryostat and tested at temperatures between 80 and 380 K. The emitted radiation was collected with a f/1 off-axis parabolic mirror and then analyzed using a Fourier transform infrared spectrometer (FTIR) Bruker Vertex 70 equipped with a pyroelectric detector. For optical power measurements, the laser beam was collimated with another f/1 off-axis parabolic mirror onto a Melles Griot 13PEM001 power meter. The lasers were tested in pulsed mode using 333-ns-long current pulses at a repetition rate of 12 kHz. An additional 30 Hz current modulation was applied for measurements of light-current curves with the slow FTIR detector. The pulsed optical power of the lasers was calibrated using the power meter at a 5% duty cycle (100 ns/500 kHz). No correction was made to take into account the collection efficiency and other optical loss in the experimental setup.
